# The possibility of patient-centered dietetic intervention in the context of health literacy in Hungary

**DOI:** 10.3389/fnut.2025.1668048

**Published:** 2026-01-06

**Authors:** Zsuzsanna Szucs, Ida Ercsey, Anita Horkai, Helga Judit Feith

**Affiliations:** 1 Division of Health Sciences, Doctoral College, Semmelweis University, Budapest, Hungary; 2 Faculty of Marketing and Management, Széchenyi István University, Győr, Hungary; 3 Department of Social Sciences, Faculty of Health Sciences, Semmelweis University, Budapest, Hungary

**Keywords:** health literacy, medical nutrition therapy, dietetic care, patient-centered care, nutrition education, food-based dietary guidelines

## Abstract

**Introduction:**

Non-communicable diseases (NCDs) accounted for 74% of global deaths in 2024, with over 41 million people dying. The WHO has identified reducing behavioral and metabolic risk factors as a priority intervention. In modern healthcare, patient-centered care plays a key role by addressing individual needs, lifestyles, and motivations, thereby enhancing the effectiveness of prevention and behavior change. Food-based dietary guidelines (FBDG), such as Hungary’s OKOSTÁNYÉR^®^, are vital in prevention and medical nutrition therapy. However, the effectiveness of such dietary interventions largely depends on individuals’ health literacy.

**Objectives:**

This study aimed to explore the relationship between health literacy, dieting habits, dietary counseling, and awareness of the local FBDG recommendations.

**Methods:**

This cross-sectional study was conducted in February 2023 on a representative sample (*N* = 500) of the adult Hungarian population, using the CAWI method. Data were collected with the validated HLS-EU 47-item questionnaire and additional custom items on health status, dieting, and awareness of the local FBDG. Health literacy indexes were calculated using validated thresholds. Descriptive statistics, chi-square tests, ANOVA, and multivariate regression analyses were applied (*p* < 0.05).

**Results:**

A total of 77.4% of respondents had low (insufficient or problematic) health literacy. Higher education levels and younger age were significantly associated with better health literacy. Chronic diseases were more prevalent in low health literacy groups. About 32.4% of respondents followed a medically indicated diet, and 36.8% had received dietary counseling. Awareness of the local FBDG was relatively low (20.8%), particularly among men and those with lower education.

**Conclusion:**

Patient-centered care necessitates a high level of health literacy, enabling patients to actively participate in their therapy. Findings of the study highlight that low health literacy in the Hungarian population poses a major challenge to the success of dietary interventions. Targeted health communication strategies and tailored dietetic support are essential to improve the effectiveness of personalized nutrition care, particularly among vulnerable groups with limited health literacy.

## 1 Introduction

In 2024, chronic non-communicable diseases were responsible for an estimated 41 million deaths worldwide, accounting for 74% of all global deaths. Over 15 million of these deaths occurred among individuals aged 30 to 69 years. Cardiovascular diseases accounted for the highest number of deaths, followed by cancers, respiratory diseases, and diabetes. The causes of chronic non-communicable diseases are varied, stemming from a combination of genetic, physiological, environmental, and behavioral factors, including unhealthy diets and metabolic issues such as obesity. To mitigate the health, economic, and social burdens associated with these diseases, the World Health Organization (WHO) identifies reducing risk factors as a priority intervention area (1).

Food-based dietary guidelines (FBDG), represented in Hungary by the OKOSTÁNYÉR (Smart Plate)^®^. Nutrition Guidelines, (2, 3) aim to ensure optimal energy and nutrient intake at the population level. They guide individuals toward healthier eating habits by providing practical advice on food choices, food groups, and dietary patterns. Dietary recommendations are a crucial element of dietary interventions that can help reduce the risk of chronic non-communicable diseases and inform their dietary therapy through various means, including public education, national public health programs, and modifying the food environment (2, 4).

However, recognizing the considerable diversity in individual nutritional needs, professional guidelines stress the importance of personalized approaches to dietary therapy (5–7). These approaches must assess and consider individual preferences, needs, and values. Patient-centered care (PCC) addresses these needs by ensuring that an individual’s values guide all clinical decisions, including those related to dietetic care (8, 9). Furthermore, an individual’s ability to acquire, understand, process, and apply health-related information, such as knowledge about healthy eating, is a fundamental determinant of the effectiveness of nutrition interventions (10).

Based on health literacy studies conducted in Hungary (11–14), it is likely that a significant portion of the target population for dietary interventions can only comprehend and act upon information and guidance, such as recommended dietary practices, with assistance. Recent studies have highlighted a significant prevalence of low health literacy (11, 12) among the Hungarian population, revealing concerning levels of both functional (14) and practical health literacy (13). These studies affirm the previously established connection between low health literacy and the development of chronic diseases. However, to date, there has been no research conducted in Hungary or elsewhere that specifically examines health literacy in relation to the presence of chronic diseases, dietary practices, consultations with dietitians, or awareness of local nutritional recommendations.

Patient-centered care, which benefits therapeutic outcomes and quality of life, is based on enhancing the patient’s self-management skills and competencies (9, 15, 16). This approach requires health professionals, including dietitians, to possess a solid understanding of the population’s health literacy. They must convey messages about nutrition therapy using effective communication tools that are tailored to the specific needs of the target audience.

This study aims to explore the relationship between health literacy, dieting, dietitian advice, and knowledge of the local FBDG. While the examination of nutritional habits, nutrient intake, and dietary adherence was not the primary focus of our research, it does suggest promising avenues for future investigation.

## 2 Materials and method

This survey was conducted in February 2023 with a representative sample of the Hungarian adult population, stratified by age, sex, and place of residence (*N* = 500). The data collection was carried out using the CAWI (Computer Assisted Web Interview) method, which allowed for online interviews.

The study employed the validated HLS-EU 47 questionnaire (17) that included demographic questions and assessed health literacy in three different domains: (1) health system-related literacy, (2) disease prevention, and (3) health promotion. Health literacy indices were defined using the methodology developed by the HLS-EU consortium, adhering to internationally validated thresholds (18). The European Health Literacy Survey (HLS-EU) was developed following the methodology outlined by Sørensen et al. (17). In the HLS-EU survey, 13 index thresholds were established in line with standard practices for measuring health literacy. These thresholds were designed to ensure that the correlation patterns between health literacy levels and significant covariates differed only minimally from the metric health literacy scores, while maximizing the correlation between the levels and the metric scores. The resulting four categories were defined as follows: “insufficient” (0–25), “problematic” (> 25–33), “sufficient” (> 33–42), and “excellent” (> 42–50) health literacy (18).

This study successfully applies the overall index and its three sub-indices to the Hungarian sample, and the analysis of the scale’s internal consistency confirms its reliability. The Cronbach Alpha values are particularly high, exceeding 0.9 for all indices. The Cronbach-α value was considered, which quantifies internal consistency, to be acceptable if it is between 0.70 and 0.96. The reliability of the overall health literacy index is confirmed by the Cronbach’s alpha value of 0.960. The validated health literacy scale contains 3 dimensions. The health subscale is measured with 16 items on a 1–4-point scale. The internal consistency of the subscale is confirmed by the high Cronbach’s alpha value of 0.904. Health prevention is measured using a 15-item subscale, whose internal consistency is reliable, with a Cronbach’s alpha value of 0.901. The third subscale is health development, for which 16 items were used. The reliability of the subscale is supported by the value of the coefficient 0.919.

Descriptive statistical indicators, such as mean, standard deviation, and frequency, were calculated to characterize the variables in the quantitative research. Bivariate statistical methods were utilized to examine the relationships between independent and dependent variables. The chi-squared test was used to explore correlations among categorical variables, including socio-demographic characteristics and specific health determinants (such as chronic diseases, following a diet, nutritional advice received from a dietitian, and knowledge of the local FBDG) concerning health literacy levels. The variables and measurement tools used in the study are listed in Table 1.

**TABLE 1:** The variables and measurement tools used in the study.

Variables	Measure			
Gender	1 = male 2 = female			
Age	Age in years			
Level of education	1 = primary school to 5 = tertiary education degree			
Place of residency	Name and postal code of the settlement			
Chronic illnesses	Do you have any chronic illnesses? 1 = yes 2 = no			
Diet	Are you on a diet due to any health problems? 1 = yes 2 = no			
Nutritional advice	Have you ever received nutritional advice from a dietitian? 1 = yes 2 = no			
Knowledge of OKOSTÁNYÉR^®^	Are you familiar with the Hungarian nutritional recommendation, OKOSTÁNYÉR^®^? 1 = yes 2 = no			
**How easy or difficult it is.**	**Very difficult**	**Difficult**	**Easy**	**Very easy**
Health care	…find information about symptoms of illnesses that concern you?			
	…find information on treatments of illnesses that concern you?			
	…find out what to do in case of a medical emergency?			
	…find out where to get professional help (such as doctor, pharmacist, psychologist) when you are ill?			
	…understand what your doctor says to you?			
	…understand the leaflets that come with your medicine?			
	…understand what to do in a medical emergency?			
	…understand your doctor’s or pharmacist’s instruction on how to take a prescribed medicine?			
	…judge how information from your doctor applies to you?			
	…judge the advantages and disadvantages of different treatment options?			
	…judge when you may need to get a second opinion from another doctor?			
	…judge if the information about illness in the media (such as TV, internet, or other media) is reliable?			
	…use information the doctor gives you to make decisions about your illness?			
	…follow the instructions on medication?			
	…call an ambulance in an emergency?			
	…follow instructions from your doctor or pharmacist?			
Disease prevention	…find information about how to manage unhealthy behavior such as smoking, low physical activity and drinking too much?			
	…find information on how to manage mental health problems like stress or depression?			
	…find information about vaccinations and health screenings (such as breast exam, blood sugar test, blood pressure) that you should have?			
	…find information on how to prevent or manage conditions like being overweight, high blood pressure or high cholesterol?			
	…understand health warnings about behavior such as smoking, low physical activity and drinking too much?			
	…understand why you need vaccinations?			
	…understand why you need health screenings (such as breast exam, blood sugar test, blood pressure)?			
	…judge how reliable health warnings are, such as smoking, low physical activity and drinking too much?			
	…judge when you need to go to a doctor for a check-up?			
	…judge which vaccinations you may need?			
	…judge which health screenings (such as breast exam, blood sugar test, blood pressure) you should have?			
	…judge if the information on health risks in the media (such as TV, Internet or other media) is reliable?			
	…decide if you should have a flu vaccination?			
	…decide how you can protect yourself from illness based on advice from family and friends?			
	…decide how you can protect yourself from illness based on information in the media (such as Newspaper, leaflets, Internet or other media)?			
Health promotion	…find information on healthy activities such as exercise, healthy food and nutrition?			
	…find out about activities (such as meditation, exercise, walking, Pilates etc.) that are good for your mental wellbeing?			
	…find information (such as reducing noise and pollution, creating green spaces, leisure facilities) on how your neighborhood could be more health-friendly?			
	…find out about political changes (such as legislation, new health screening programs, change of government, restructuring of health services etc.) that may affect health?			
	…find out about efforts to promote your health at work?			
	…understand advice on health from family members or friends?			
	…understand information on food packaging?			
	…understand information in the media (such as Internet, newspaper, magazines) on how to get healthier?			
	…understand information on how to keep your mind healthy?			
	…judge how where you live (such as your community, neighborhood) affects your health and wellbeing?			
	…judge how your housing conditions help you to stay healthy?			
	…judge which everyday behavior (such as drinking and eating habits, exercise etc.) is related to your health?			
	…make decisions to improve your health?			
	… join a sports club or exercise class if you want to?			
	…influence your living conditions that affect your health and wellbeing?			
	…take part in activities that improve health and wellbeing in your community?			

A one-way analysis of variance (ANOVA) was conducted to investigate the effect of nominal variables with two or more categories on metric variables. The assumptions for ANOVA, including normality and homogeneity of variance, were verified using Levene’s test. This analysis was appropriate for determining differences in scores across health literacy sub-indices between the two health literacy clusters (limited and adequate health literacy).

Multivariate regression models were employed to assess the impact of explanatory and control variables on the development of health literacy sub-indices and the overall health literacy index. These regression models were evaluated using F-tests, while individual regression coefficients were tested with *t*-tests. The condition of multivariate regression analysis—specifically, the absence of multicollinearity—was assessed through the correlation coefficients presented in the correlation matrix.

Results were deemed significant at *p* < 0.05, with a 95% confidence interval. Data were recorded and processed using SPSS version 27.0.

Participation in the online study was voluntary and contingent upon signing a consent form, following verbal and written information provided to participants. The study adhered to the principles outlined in the Declaration of Helsinki. Ethical clearance for the study was obtained from the Medical Research Council (Reference Number: BMEÜ/333-3/2022/EKU).

## 3 Results

### 3.1 Characterization of the study population

Almost half of the participants in the survey are male (47.0%), while the majority are female (53.0%). In terms of age distribution, the sample includes 113 individuals aged 65 and over (22.6%), followed by those aged 40–49 at 19.0%. The age groups of 18–29 and 30–39 comprise 87 (17.4%) and 85 (17.0%) participants, respectively. The 50–59 age group accounts for 75 respondents (15.0%), while the 60–64 age group has 45 respondents (9.0%). The average age of the respondents is 48.3 years, with a standard deviation of 17.2 years.

In terms of education, 43.0% of the respondents have a secondary education, 41.6% have a tertiary education, and 11.8% have only completed primary education. Approximately one-third (32.6%) of respondents live in towns smaller than the county capitals. Pest County has the highest number of respondents (159), and nearly one-third (31.8%) of the sample is from the Central-Hungary region, which contributes to the overall demographic data from seven regions. Table 2 summarizes the socio-economic characteristics of the sample.

**TABLE 2:** Summary of socio-demographic characteristics of the study sample (own editing).

Variable	Category	Ratio (%)
Gender	Male	47.0
	Female	53.0
Age	18–29 years	17.4
	30–39 years	17.0
	40–49 years	19.0
	50–59 years	15.0
	60–64 years	9.0
	65 years+	22.6
Level of education	Primary school	3.6
	Secondary school (w/o certificate)	11.8
	Secondary school (w certificate)	43.0
	Tertiary education degree	41.6
Place of residency (type)	Budapest	20.0
	County seat	22.2
	City	32.6
	Village	25.2
Place of residency (region)	Southern Great Plain	12.2
	Southern Transdanubia	9.4
	Northern Great Plain	15.4
	North-Hungary	11.8
	Central Transdanubia	10.8
	Central-Hungary	31.8
	Western Transdanubia	8.6

### 3.2 Determinants of health literacy

In the first part of the study, health literacy of the population was examined; their skills and abilities to acquire, understand, process, and apply health information.

**FIGURE 1:**
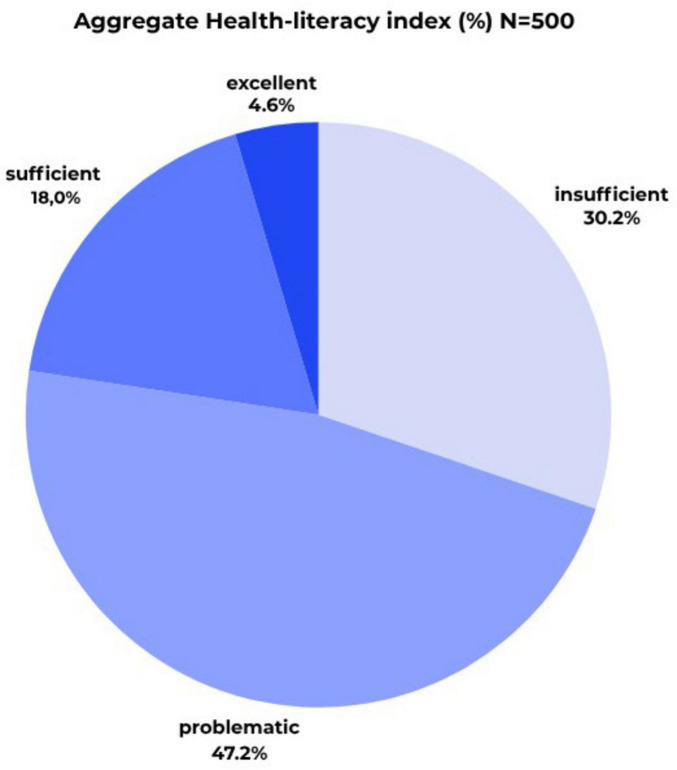
Aggregate health literacy index (%) (*N* = 500) (own editing).

Based on the validated thresholds, the study sample showed that 4.6% were classified as having excellent health literacy, 18.0% as sufficient, 47.2% as problematic, and 30.2% as having insufficient health literacy (Figure 1).

Multivariate regression analysis was employed to examine the explanatory and control variables associated with the development of health literacy sub-indices and the overall health literacy index. In this model, dietary adherence (yes/no) served as the explanatory variable, while age, education, and place of residence (region) acted as control variables. The inclusion of control variables is justified since socio-demographic factors significantly impact health outcomes in the studied populations. These control variables were measured as ordinal and nominal data and were transformed into dummy variables suitable for regression analysis.

Respondents’ ages were categorized into six groups, with the age group of 18–29 years designated as the reference. Education levels were classified as primary, secondary, and tertiary, with primary education serving as the baseline for interpreting the results. Among the seven regions in Hungary, the Southern Great Plain Region was selected as the reference point.

**FIGURE 2:**
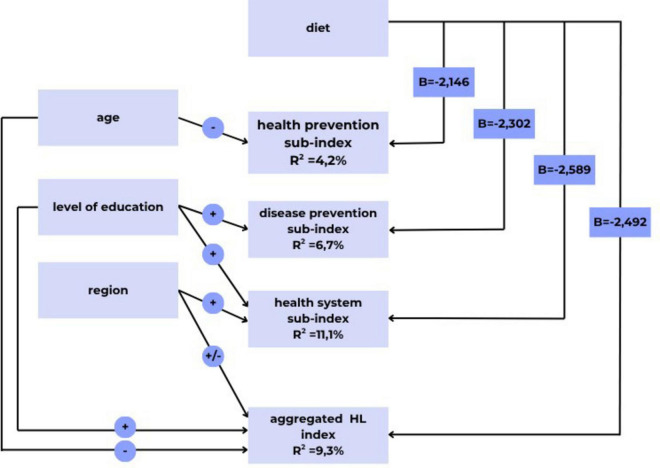
Significant results of multivariate regression analysis (own editing). *p* < 0.05. *R*^2^—Multiple coefficient of determination. What percentage of the variance in the dependent variable (e.g., health promotion sub-index) is explained by the independent variables (e.g., age, diet). B–Regression coefficient. A negative B coefficient value is the difference between the health literacy levels of dieters and non-dieters. ±: Indicates a positive (+) or negative (–) change in the value of the health literacy sub-indices and the overall health literacy index, relative to the reference group of control variables.

The significant findings from the multivariate regression analyses are summarized in Figure 2. In this figure, the effects of the control variables are indicated by positive (+) or negative (−) signs, representing the changes in the values of the three health literacy sub-indices and the overall health literacy index relative to the reference groups for each control variable. The impact of dietary adherence as an explanatory variable is reflected in the non-standardized regression coefficient (B). A negative B coefficient indicates a difference in health literacy between dieters and non-dieters.

Separate regression analyses were conducted for the aggregate health literacy index and the three sub-indices. The index scores of the dependent variables (three health literacy sub-indices and the aggregate health literacy index) were utilized in the regression models. Initially, we reviewed the descriptive statistics (mean and standard deviation) of the dependent variables, relative to a maximum score of 50. The overall health understanding index score was found to be 28.4 ± 7.0 points, for the health system sub-index 28.87 ± 7.38 points, for the prevention sub-index 28.59 ± 7.66 points, and for the health promotion sub-index 27.63 ± 8.26 points.

In the regression model of the aggregate health literacy index, dietary intake was found to have a significant effect on health literacy, alongside the three control variables (age, education, and region), accounting for an explanatory power of almost 10% (*R*^2^ = 9.3%).

Among the health literacy sub-indices, the explanatory and control variables included in the study account for a significant portion (11.1%) of the variation in the average score for the health system. This suggests that when controlling for respondents’ education and region of residence, diet becomes an important factor influencing the variations in the health system sub-index values.

In contrast, the health understanding sub-index related to prevention is less influenced by the independent variables (*R*^2^ = 6.7%), even when accounting for education level and dietary habits. In the area of health promotion, only age and diet control significantly impacted the health understanding sub-index, although the explanatory power was relatively low (4.2%).

Educational attainment showed a notable association with domains such as prevention, the health care system, and overall health literacy. Specifically, respondents with secondary and tertiary education exhibited higher health literacy compared to those with only primary education, as illustrated by the positive (+) notation in the graph. Age also played a significant role in health promotion and overall health understanding. Within the age categories, the 18–29 group served as the reference group, and those aged 60–64 and 65 and older demonstrated lower (−) health literacy in health promotion.

The region of residence is also a significant factor influencing the average health system score and the overall health literacy index. In this study, we examined diet-related factors, revealing that respondents’ awareness of the dietary guidelines significantly impacted their average health understanding scores across all domains. The results indicate that individuals on specific diets tended to have lower health literacy, characterized by a negative B coefficient value. The most considerable difference (*B* = −2.589) was observed in the health system domain, while the smallest difference (*B* = −2.146) was recorded for health promotion.

### 3.3 Differences between sub-indices

In the analysis, the sample was divided into two clusters: (1) the insufficient and problematic group, referred to as limited health literacy (LH), and (2) the sufficient and excellent group, referred to as adequate health literacy (AH). This clustering was necessary to examine differences in each sub-index based on distinct populations.

For the health system sub-index, both clusters recorded the lowest scores in terms of the perception of whether information about illness obtained from the media is reliable (AH = 2.89; LH = 2.10). This perception is particularly challenging for those with limited health literacy. The analysis of variance shows that the difference between the two clusters is significant (*F* = 105.4; *p* < 0.001). With an Eta^2^ value of 0.175, we conclude that the differing levels of health literacy in the two clusters affect the perception of the reliability of media-derived disease information by 17.5%.

A similar trend is observed in the disease prevention sub-index. Respondents with limited health literacy exhibited the most significant difficulty in assessing the reliability of information on health risks obtained from the media (LH = 2.11; AH = 2.96). Again, there is a significant difference in the evaluations between the two clusters (*F* = 119.9; *p* < 0.001). In the area of disease prevention, the disparity in health literacy between the limited and adequate clusters results in a 19.4% difference in perceived reliability of media information regarding health risks.

In the health promotion sub-index, the most substantial difference between the two clusters was noted in decision-making to improve health (AH = 3.27; LH = 2.35). This difference is significant (*F* = 146.1; *p* < 0.001), indicating that the variation in health understanding between the clusters affects respondents’ ability to make health-related decisions by 22.7%. Additionally, the limited health literacy cluster faces significant challenges in influencing their living conditions, which, in turn, greatly impacts their health. Among these respondents, this factor scored the lowest (2.25).

### 3.4 Chronic disease, dieting, nutrition recommendations

This study examined several factors that affect respondents’ health, including the presence of chronic illness, dieting, participation in nutritional counseling, and knowledge of the local dietary guideline.

**FIGURE 3:**
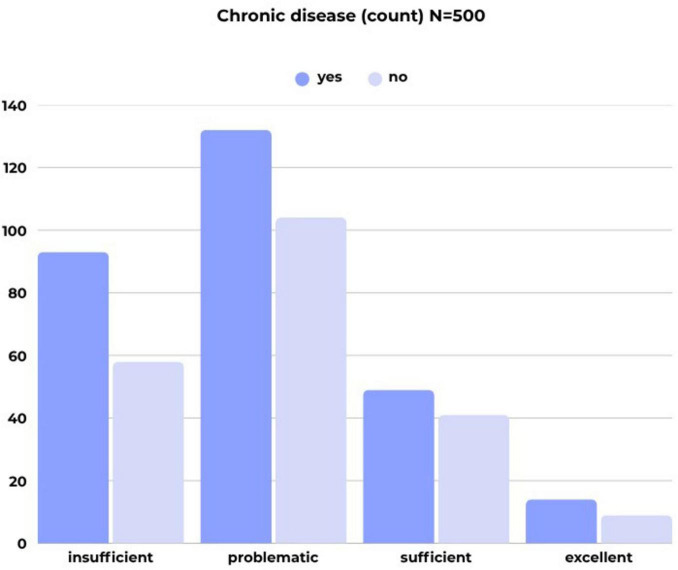
Presence of chronic disease by health literacy category (count; *N* = 500) (own editing).

Over half of the respondents reported having a chronic disease. Notably, a higher percentage of respondents in the older age groups—specifically those aged 60–64 years (80.0%) and 65 years and older (80.5%)—were found to have chronic illnesses. The correlation between age and the presence of chronic disease is significant, although the strength of the association is considered weak (*C* = 0.323). Additionally, the prevalence of chronic diseases was found to be higher among individuals with limited health literacy, as clearly illustrated in Figure 3.

Approximately one third (32.4%) of survey participants are currently on a diet due to a health issue. Additionally, a greater number of respondents (184, or 36.8%) reported having received dietary advice from a dietitian in the past.

The sample shows a relatively low level of awareness regarding the local FBDG, with only one fifth of respondents (20.8%) indicating that they are familiar with it. Gender, age, and education level were associated with knowledge of the dietary recommendations. Female respondents exhibited a higher level of awareness (25.7%) compared to males and the overall sample (20.8%). This association is significant (*p* = 0.004), although the strength of the association is relatively weak (*C* = 0.126).

Respondents aged 30–39 and 40–49 showed higher levels of knowledge about the Hungarian FBDG, with rates of 23.5% and 24.2%, respectively, compared to other age groups. Although the correlation between age and knowledge is not statistically significant, a visible trend exists. Among graduates, awareness of the local dietary recommendations was higher (25.5%) than among those with lower levels of education (13.6% and 18.6%). Again, while the correlation between educational attainment and knowledge of the recommendation is not statistically significant, a trend can be observed.

**FIGURE 4:**
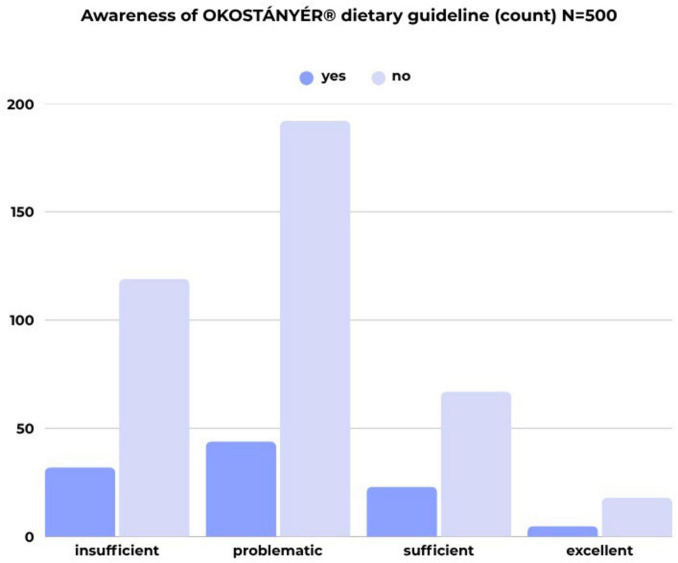
Awareness of local nutritional recommendations by health literacy category (count; *N* = 500) (own editing).

Most of the respondents (104) who were aware of the FBDG belonged to the group of insufficient (44) or problematic (32) health literacy (Figure 4).

Relationships between having a chronic disease, dieting, receiving dietary advice from a dietitian were analyzed, alongside with the awareness of the local dietary recommendations. The results indicate a significant association between having a chronic disease and dieting (*p* < 0.001). Among respondents with a chronic disease, a higher proportion (44.4%) reported following a diet. However, the strength of this relationship is considered weak to moderate (*C* = 0.287).

Additionally, there is a noticeable trend among those with chronic diseases, where 23.6% reported being aware of the FBDG, compared to 20.8% in the overall sample.

This study also found a significant correlation (*p* < 0.001) between having a chronic disease and seeking nutritional advice. Among individuals with chronic diseases, a greater percentage (46.5%) consult a dietitian for dietary guidance. The strength of this association is also categorized as weak to moderate (Phi = 0.235).

## 4 Discussion

The importance of nutrition in enhancing health and quality of life, as well as in preventing and managing chronic diseases, is clear. Maintaining a healthy dietary pattern throughout one’s life helps prevent malnutrition and its various forms, which include non-communicable diseases such as obesity, type 2 diabetes, and cardiovascular diseases. While the basic principles of a varied, balanced, sustainable, and healthy diet remain consistent, individual nutritional needs can differ based on personal characteristics (1, 19, 20). FBDGs are designed to promote optimal energy and nutrient intake at the population level; however, they should also be personalized to address specific dietary needs for disease prevention or medical nutrition therapy (5).

PCC acknowledges and respects the unique needs, values, preferences, and socio-ecological and psychological characteristics of individuals, while positioning them at the core of healthcare (21). The patient-centered approach can enhance the quality of collaboration between patients and dietitians and build trust in healthcare professionals. As a result, it can lead to improved adherence to treatment and a better quality of life for patients (22).

PCC builds on the patient’s capability for informed and responsible decision-making, their ability to effectively self-manage and cope with the therapy. For patient-centered care to be a reality, patients must acquire the skills necessary for effective collaboration in which improvement of health literacy plays a key role (10, 21).

Results of this research indicate that the health literacy level among the Hungarian adult population presents significant gaps, which may serve as a major barrier to the prevention and management of chronic diseases. Over three-quarters of the sample (77.4%) can be classified as having “inadequate” or “problematic” health literacy, confirming the assumption that a substantial portion of the population struggles to interpret and apply health information. These findings align with other national and international studies that have reported similarly low levels of health literacy (11–14, 18, 23).

In the present study, the explanatory power of the independent variables included in the analysis is relatively small. Therefore, it is worthwhile comparing the results with those of previous studies, (12, 18) where the same method was used. Based on the Hungarian results, 15 percent of health literacy was explained by the socio-demographic explanatory variables built into the model (gender, age, educational attainment, material deprivation and social status). From an international perspective, higher explanatory power was found in Bulgaria (25%), Greece (29%), and Poland (21%). This figure was somewhat lower in Ireland (19%) and even weaker in Austria (10%), Germany (8%), Spain (9%) and the Netherlands (8%).

Regarding factors influencing health literacy, educational attainment demonstrated a notable association with domains such as prevention, the healthcare system, and overall health literacy. Respondents with higher levels of education exhibited higher health literacy compared to those with only primary education. Our research findings align with previous studies, both domestic (11–14) and international (18, 23), indicating that low educational attainment correlates with lower health literacy. The previous studies (12, 18) found that higher levels of health literacy were associated with being female, being younger, having a higher level of education and having a higher income. In the linear regression model, the effect of the independent variables on overall health literacy was expressed using standardized regression coefficients. For Hungarian respondents, the effects were as follows: gender, 0.08; age, −0.10; educational attainment, 0.15; and financial deprivation, −0.24. In an international comparison, the coefficient value for age ranged from 0.09 to 0.17. In countries where age was a significant explanatory variable, younger people had a higher health perception. However, age does not affect health perception in Germany, Ireland and the Netherlands. The regression coefficients showing the effect of educational attainment in the eight countries included in the study range from β = 0.08 to β = 0.22. Increased educational attainment increases health literacy in all countries except Austria and Ireland, where no significant relationship was found between the two factors.

This highlights the crucial role of education in mitigating social inequalities, which includes addressing health literacy disparities. The early integration of knowledge related to health (including diet and lifestyle choices), into school education in a more explicit and practice-oriented manner can be an effective tool for improving health literacy, maintaining health, reducing chronic diseases, and enhancing cooperation with medical nutrition therapy in cases of existing diseases (24–26).

Age also showed an association with health promotion and overall health understanding, where older populations, aged 60–64 and 65 and above, demonstrated lower health literacy, which is consistent with the results of previous research (11, 14, 27, 28). Although our research did not investigate the underlying causes, previous studies (28–30) have identified several factors behind this phenomenon, such as social (e.g., lack of social support, isolation), sociodemographic (e.g., advanced age), economic (e.g., lower income) and health aspects (e.g., chronic conditions, reduced mobility, mental health challenges). A cross-sectional literature review was conducted by Battineni et al. (30) investigating factors affecting the quality and reliability of online health information. They found that older adults prefer direct visits with doctors and healthcare professionals and are less interested in accessing online health information. Poor health literacy among older adults is a complex issue that involves not only communication and information acquisition but also requires a multifaceted solution. This solution must involve public health programs as part of a comprehensive national health promotion strategy. Health professionals, including dietitians, need to develop the skills necessary to educate older adults with low health literacy effectively. Furthermore, when providing nutritional recommendations, it is essential to create approaches that are tailored to inform vulnerable individuals with low health literacy, focusing on improving the method of delivery, presentation, and clarity of wording.

An analysis of sub-indices revealed that individuals with low health literacy particularly struggle with assessing the reliability of information related to the health system and disease prevention. This is concerning, as reducing the risk of chronic diseases heavily relies on individuals recognizing and utilizing information from credible sources (31). Additionally, those in the low health literacy group exhibit a higher prevalence of chronic diseases and are less aware of the Hungarian dietary recommendations, which are crucial for prevention and dietetic communication.

The examination of sub-indices also revealed that respondents with limited health literacy experienced the most significant difficulty in assessing the reliability of health information obtained from the media. Survey data shows that sources of health-related information are increasingly shifting to digital channels. According to Eurostat data (32), the share of individuals seeking health information online has increased in almost all EU Member States over the last decade, rising by 17 percentage points in the EU from 38% (2011) to 55% (2022). In Hungary, this proportion was 65%. Technological advances have made it easier to access health information on the internet, but at the same time, they have increased the likelihood of encountering incorrect or unreliable health information. The low level of health literacy and the difficulty in assessing the reliability of health information found in the media are likely to be influenced by the issues discussed above, such as age and educational attainment, as well as lower digital skills (33). To enhance communication with patients who have low health literacy, various literature reviews highlight the significance of a multi-level approach, along with practical considerations such as language and visual presentation (34, 35). This indicates that, alongside individual therapeutic approaches like dietitian-patient consultations, we also need systemic measures involving health, education, and social systems, as well as societal measures such as public health strategies and community initiatives. Additionally, guidelines exist to enhance the quality of health information available on the internet, including the Suitability Assessment of Material guidelines and relevant literature reviews (36, 37). Which supports healthcare professionals in developing digital educative content for the general public.

The significant connection between the presence of chronic diseases and dietary adherence suggests that some individuals are more motivated to adopt lifestyle changes following a diagnosis. However, this study did not delve into this particular issue. This finding aligns with previous research (38), which indicates a lack of a preventive approach in Hungary, as a greater proportion of patients consult a dietitian only after a disease has been established, mainly upon the recommendation of a general practitioner or specialist.

Moreover, it is evident that the success of lifestyle changes and nutritional therapy is largely dependent on the individual’s level of health literacy. While chronic patients are more likely to seek dietary advice, a lack of skills in interpreting and applying this information can hinder effective intervention. The authors must acknowledge certain limitations of the study. The HLS-EU-Q47 tool assesses the perceived difficulty of a selected health task using a 4-point self-assessment scale (very easy, easy, difficult, and very difficult). This tool measures an individual’s health literacy by reflecting the balance between their personal competencies and the complexity or demands of various situations. It’s important to note that local factors, such as the healthcare system or patient pathways, can influence these perceptions. Therefore, when interpreting survey results, especially in international comparisons, these factors should be considered.

For collecting data, the CAWI is included in the HLS19 Study Protocol as being acceptable method (39). The data collection was carried out using an established online panel, which may lead to an overrepresentation of individuals with higher educational attainment and greater digital activity. This is evident in the sample, with individuals with low educational attainment making up only 3.6% of the total. Another limitation is the lack of weighting of the sample for population density (e.g., ethnicity). Furthermore, due to the cross-sectional design, causal relationships cannot be established.

The study has a significant strength: it is the first to explore the relationships between health literacy, dieting, the use of dietetic counseling, and awareness of the local food-based dietary guidelines (FBDG) within the Hungarian adult population, therefore offers valuable and new insights that can help improve dietetic practice.

## 5 Conclusion

Results of this study highlight the importance of PCC and personalized dietary approaches. If we do not consider the differences in health literacy among various populations, the effectiveness of nutrition counseling may be limited. The WHO emphasizes a collective responsibility for all information providers, including decision-makers, civil society, and health services, to ensure access to reliable health information that is comprehensible and actionable for everyone (10). Findings of this research can enhance dietary care in several ways. The first step in the dietetic intervention, known as the Nutrition Care Process (NCP), is the Nutrition Assessment (40). This step aims to evaluate an individual’s nutritional status by interpreting clinical information, which includes their medical history, dietary habits, and physical measurements. During this process, it would be beneficial to gather information about the individual’s health literacy. This can improve the effectiveness of medical nutrition therapy and patient education.

In healthcare, systematically screening for health literacy using appropriate tools can provide valuable insights for healthcare professionals. However, if screening is not feasible, professionals should still consider the risk of low health literacy among high-risk patients, such as those with low educational attainment, older individuals, or those from low socioeconomic backgrounds, and incorporate this into their care strategies. The concept of patient-centered dietary care, which takes into account the specific characteristics of health literacy, should be incorporated into healthcare guidelines and professional training curricula. It is essential to continuously review and develop dietary guidelines, including creating educational materials and communication tools (such as infographics and audiovisual content) that effectively reach populations with low health literacy.

The knowledge gained from our research, particularly through identifying at-risk groups, is also important for developing public health programs. This insight can contribute to creating more effective and inclusive prevention strategies.

## Data Availability

The raw data supporting the conclusions of this article will be made available by the authors, without undue reservation.
